# Production of 2,3-butanediol from cellulose and *Jatropha* hulls after ionic liquid pretreatment and dilute-acid hydrolysis

**DOI:** 10.1186/2191-0855-3-48

**Published:** 2013-08-20

**Authors:** Li-qun Jiang, Zhen Fang, Xing-Kang Li, Jia Luo

**Affiliations:** 1 Biomass Group, Laboratory of Tropical Plant Resource Science, Xishuangbanna Tropical Botanical Garden, Chinese Academy of Sciences, 88 Xuefulu, Kunming, Yunnan Province 650223, China; 2 University of Chinese Academy of Sciences, 19A Yuquan Road, Beijing 100049, China

**Keywords:** Ionic liquid, Hydrolysis, 2,3-Butanediol, Fermentation, *Klebsiella oxytoca*, *Jatropha* hulls

## Abstract

Abundant *Jatropha* waste is a promising renewable feedstock for the production of sugars and 2,3-butanediol fermentation. To obtain high yield of water-soluble products and high concentration of reducing-sugars, ionic liquid (IL) pretreatment and dilute acid hydrolysis at 150°C were combined in this work. The destruction of crystalline structure and increase surface area of biomasses after IL-pretreatment, made their hydrolysis more efficient*.* Compared with original cellulose, after IL-pretreatment, both the yield and concentration of reducing-sugars increased by 139%, and the water-soluble products yield increased by 128% after hydrolysis. Compared with water-washed *Jatropha* hulls, after IL-pretreatment, the yield and concentration of reducing-sugars increased by 80% and 76%, respectively, and the water-soluble products yield increased by 70% after hydrolysis. IL-pretreatment benefited the fermentation of *Jatropha* hull hydrolysate with 66.58% diol yield and its productivity increased from 0.35 to 0.40 g/(L · h).

## Introduction

2,3-Butanediol is a promising liquid fuel and bulk chemical for comprehensive industry applications (Garg and Jain 1995; Syu 2001). Biological production of 2,3-butanediol on an industrial-scale is still in its early stage but with strong prospects of growth. However, the substrates account for more than half of the total production cost, and strongly influence the economy of its production (Celińska and Grajek 2009; Wang et al. 2010; Ji et al. 2011). As the most abundant, cheap and renewable source of sugars substrate, lignocellulose is a promising feedstock for biorefinery. *Jatropha* hulls are wastes from *Jatropha* seeds for biodiesel synthesis. One tonne of *Jatropha* seeds provides about 350-L crude oil for biodiesel production leaving 2.4 tonne hulls as waste (Sharma et al. 2009). As *Jatropha* hulls are rich in carbohydrates, studies are required to efficiently utilize these polysaccharides to produce sugars for fermentation.

Fermentable sugars can be produced by pretreatment and subsequent enzymatic hydrolysis. In previous study (Marasabessy et al. 2012), a dilute acid pretreatment of *Jatropha* hulls at optimum conditions (0.9% sulfuric acid, 30 min, 178°C) for enzymatic hydrolysis and ethanol fermentation resulted in 29% pentose degradation into furfural and 5% hexose degradation into 5-hydroxymethylfurfural (5-HMF). The degradation of sugars could not only result in low yield and high cost of sugars derived from biomass, but also lead to the formation of toxic by-products for fermentation. In addition, slow hydrolysis rate, high cost of enzyme and sensitivity to contaminants originated from other biomass components restrict its economical feasibility (Brinder et al. 2010). Two-step dilute sulfuric acid hydrolysis was used to effectively hydrolyze *Jatropha* hulls at 150°C for 2,3-butanediol fermentation in our previous work (Jiang et al. 2012). Hemicellulose and amorphous cellulose of *Jatropha* hulls can be easily hydrolyzed without any pretreatment but crystalline cellulose is more difficult to be hydrolyzed, thus harsh conditions are needed for it. However, severe conditions also accelerate the secondary decomposition of sugars. In addition, low concentration of fermentable sugars in the hydrolysate is one of the critical issues in the utilization of lignocellulose for biofuels and bio-based chemicals production. Cellulose crystallinity is a negative factor that affects biomass hydrolysis. Ionic liquids (ILs) have excellent properties to treat lignocellulose and make the crystallinity remarkable decrease and structure essentially amorphous and porous for efficient hydrolysis (Tian et al. 2011; Li et al. 2013). ILs are recyclable and IL-pretreatment is considered as an environmentally-friendly alternative to conventional pretreatment methods (Liu et al. 2012; Shafiei et al. 2013). As IL-pretreatment can effectively decrystallize cellulose, both hemicellulose and cellulose components can be hydrolyzed simultaneously at relatively moderate conditions after IL-pretreated. This strategy seems a promising route to make full utilization of raw material and achieve high concentration of sugars in the hydrolysate of *Jatropha* hulls.

In this study, the feasibility of combination of IL-pretreatment with dilute acid-hydrolysis of *Jatropha* hulls for fermentable sugars to produce 2,3-butanediol was evaluated. Microcrystalline cellulose and *Jatropha* hulls were pretreated by IL1-butyl-3-methylimidazoliuma chloride ([BMIM]Cl) before their subsequent dilute sulfuric acid hydrolysis. Untreated cellulose and water-washed *Jatropha* hulls were employed as control samples for comparison. The hydrolysates were further fermented to 2,3-butanediol with *Klebsiella oxytoca*.

## Materials and methods

### Material

Original *Jatropha* hulls (OJH) were obtained from Xishuangbanna Tropical Botanical Garden in Yunnan, China. They were air-dried at 70°C for 24 h, milled then passed through 80–150 meshes. Structural carbohydrates and lignin in *Jatropha* hulls were determined according to the National Renewable Energy Laboratory (NREL) procedure (Sluiter et al. 2008). The elemental composition of *Jatropha* hulls were analyzed by an organic elemental analyzer (Vario EL III, Hanau, Germany). Microcrystalline cellulose (Cat. No. 6288) was purchased from Sigma (Shanghai), milled to the size of less than 150 meshes and dried in an oven at 60°C for 24 h before use. [BMIM]Cl (purity 99%) was from Lanzhou Institute of Chemical Physics, Chinese Academy of Sciences (Lanzhou, China). The strain used for 2,3-butanediol production was *Klebsiella oxytoca* (CICC 22912) from China Center of Industrial Culture Collection (Beijing).

### IL-pretreatment

Biomass suspension in IL[BMIM]Cl was prepared by adding 4-g (4 wt%) cellulose or OJH in a 500-mL flask containing 100-g IL. Then, without removing air, the flask was sealed with a cork and placed in oil bath at 120°C with stirring at 400 rpm for 1 h. Deionized water at 90°C (400-mL) was added to the mixture to precipitate biomass with vigorous shaking for 10 s. The regenerated biomass was then transferred into a 1000-mL beaker with deionized water at 75°C, washed with 75°C water (700-mL) thoroughly for 15 times to remove IL, freeze-dried (EYELA 1200 freeze dryer, Tokyo Rikakikai Co., Ltd.) and recovered as IL-pretreated cellulose (ILC) or IL-pretreated *Jatropha* hulls (ILJH). The recovered biomass was weighted and used for sulfuric acid hydrolysis. OJH powders were also washed with 700-mL fresh deionized water at 75°C for 15 times, freeze-dried, and recovered as water-washed *Jatropha* hulls (WJH). Recovery rate of regenerated biomass was calculated using the following equation:

(1) $$\begin{array}{l} \text{Recovery rate of regenerated biomass}\left(\mathrm{wt}\%\right)\\ \;=\left(\text{Mass of regeneraged biomass}\right)\\ \;÷\left(\text{Mass of initial biomass}\right)\times 100\% \end{array}$$

### Crystallinity measurement

Crystallinity index (CrI) of samples was determined by X-ray diffraction (XRD) in a Rigaku TTR III X-ray diffractmeter (Tokyo). X-ray diffractometer was set at 40 kV and 200 mA. Cu radiation (λ = 1.54 Å) was scanned over diffraction angle (2θ°) of 5-50° with a step of 0.01°. CrI was determined by the equation (Segal et al. 1959):

(2) $$\mathrm{CrI}\left(\%\right)=\left(I_{002}-I_{\mathrm{am}}\right)/I_{002}\times 100\%$$

where I_002_ was the highest peak intensity at an angle of diffraction of 2θ = 22.5°, whereas I_am_ was the peak for the amorphous cellulose at 2θ = 18°.

### Measurement of specific surface area

Specific surface area of samples was determined by Bruner Emmett and Teller (BET) method (Tristar II 3020, Micromeritics Instrument Co., Ltd, Northcross, GA). Samples were degassed at 100°C for 3 h and nitrogen with relative pressure of 0.05-0.985 was applied in the analyses.

### Dilute sulfuric acid hydrolysis

The acid hydrolysis reaction was carried out in a 500-mL high pressure autoclave (FCFD05-30, Yantai Jianbang Chemical Mechanical Co. Ltd., Shandong, China). For hydrolysis of *Jatropha* hulls, 15-g WJH or ILJH powders were loaded in the autoclave containing 200-mL 1.5 wt% dilute sulfuric acid solution, and reacted at 150°C for 0.5 h. For hydrolysis of cellulose, 9 g or 15 g untreated cellulose or ILC powders were loaded in the autoclave with 200-mL 4 wt% dilute sulfuric acid, and reacted at 150°C for 1 h. Concentrations of furfural and 5-HMF were analyzed by high performance liquid chromatography (HPLC, LC-20A, Shimadzu, Tokyo) fitted with a refractive index detector and Aminex HPX-87H column (Bio-Rad, Hercules, CA) at 60°C with 0.005 M H_2_SO_4_ as mobile phase at a flow rate of 0.6 mL/min. The carbon amount in the aqueous phase was determined by total organic carbon (TOC) analyzer (TOC-5000A, Shimadzu). Concentration of reducing-sugars in hydrolysate solutions was measured by 3,5-dinitrosalicylic acid (DNS) method using an ultraviolet–visible (UV-Vis) spectrophotometer (UV 1800, Shimadzu) (Miller 1959). The yield of water-soluble products and the yield of reducing-sugars were calculated as follows:

(3) $$\begin{array}{l} \mathrm{Reducing}‒\mathrm{sugars}\;\mathrm{yield}\left(\mathrm{wt}\%\right)\\ \;=\left(\mathrm{Mass}\;\mathrm{of}\;\mathrm{reducing}‒\mathrm{sugars}\right)\\ \;÷\left(\mathrm{Mass}\;\mathrm{of}\;\mathrm{cellulose}\;+\;\mathrm{Mass}\;\mathrm{of}\;\mathrm{hemicellulose}\right)\\ \;\times 100\% \end{array}$$

(4) $$\begin{array}{l} \mathrm{Water}‒\mathrm{soluble}\;\mathrm{products}\;\mathrm{yield}\left(\mathrm{wt}\%\right)\\ \;=\left(\mathrm{Carbon}\;\mathrm{mass}\;\mathrm{of}\;\mathrm{water}‒\mathrm{soluble}\;\mathrm{organic}\;\mathrm{compounds}\right)\\ \;÷\left(\mathrm{Carbon}\;\mathrm{mass}\;\mathrm{of}\;\mathrm{biomass}\right)\times 100\% \end{array}$$

The hydrolysates were neutralized with calcium hydroxide to pH 7.0 and concentrated under vacuum at 60–70°C to achieve approximately 100 g/L sugar concentration. The concentrated hydrolysate solutions (100 mL) were detoxicated in a 250-mL flask containing 2-g activated charcoal (Baoman Biochemical Co. Ltd., Shanghai). The flask was placed in a shaker at 50°C and 200 rpm for 2 h. Finally, after removing the charcoal by filtration, the solutions were ready for fermentation.

### Fermentation

The microorganism and the media were expressed in previous study (Jiang et al. 2012). Fermentation experiments were carried out in 250-mL flasks loaded with 50-mL sample solution at 150 rpm and 37°C for 60 h. Samples were collected periodically for product concentration analyses. Dry cell weight (DCW) was determined by measuring the absorbance of broth at 600 nm (OD_600_) using an UV-Vis spectrophotometer (UV1800, Shimadzu) and calculated by the calibrated equation: DCW (g/L) = 0.4491× OD_600_ + 0.0388 (R^2^ = 0.984). The concentration of 2,3-butanediol and acetoin in fermentation broth was determined by HPLC fitted with a refractive index detector and Aminex HPX-87H column at 60°C with 0.005 M H_2_SO_4_ as mobile phase at a flow rate of 0.6 mL/min. Both 2,3-butanediol and acetoin were considered as the target products in 2,3-butanediol fermentation, and expressed as diol yield:

(5) $$\begin{array}{l} \mathrm{Diol}\;\mathrm{yield}\left(\mathrm{wt}\%\right)=\left(\mathrm{Mass}\;\mathrm{of}\;2,3‒\mathrm{butanediol}\;+\;\mathrm{Mass}\;\mathrm{of}\;\mathrm{acetoin}\right)\\ \;÷\left(\mathrm{Mass}\;\mathrm{of}\;\mathrm{consumed}\;\mathrm{reducing}‒\mathrm{sugars}\right)\times 100\% \end{array}$$

## Results

### Biomass recovery and chemical composition before and after pretreatment

The recovery rate of regenerated biomass was determined after IL-pretreatment and collection followed by freeze-drying. It was reported that the recovery rate and chemical composition were affected by sources of biomass, types of ILs, pretreatment time and temperature (Uju et al. 2012). In this work, the recovery rate of ILC was 93.9%. *Jatropha* hulls were composed of cellulose, hemicellulose, lignin, long-chain fatty acids, free phenolics and other considerable unknown impurities (Jiang et al. 2012). After washed by water or pretreated by IL, large amount of water- or IL-soluble fraction contained in *Jatropha* hulls was removed and resulted in low recovery rate of pretreated hulls (69.7% for WJH and 66.7% for ILJH) and relatively increase of biopolymers (cellulose, hemicellulose and lignin) [from 69.6% in OJH to 88.1% in WJH and 91.6% in ILJH, respectively (Table 1)].

**Table 1:** **Chemical and elemental compositions of *Jatropha *hulls before and after pretreatment**

	**OJH (wt%)**	**WJH (wt%)**	**ILJH (wt%)**
**Recovery rate:**	—	69.7	66.7
**Components:**			
Hemicellulose	12.9 ± 0.7	14.6 ± 0.2	12.9 ± 0.1
Cellulose	34.3 ± 0.6	48.5 ± 0.4	48.8 ± 0.4
Lignin	22.4 ± 0.1	25.0 ± 0.7	29.9 ± 0.7
Total	69.6	88.1	91.6
**Elements:**			
Carbon	39.2	40.0	40.0
Hydrogen	5.5	5.0	5.9
Nitrogen	1.1	1.0	1.3
Total	45.8	46.0	47.2

The chemical components and elemental composition of untreated and pretreated *Jatropha* hulls were analyzed and listed in Table 1. The content of cellulose in ILJH (48.8%) was nearly equivalent that in WJH (48.5%), but the content of hemicellulose in ILJH (12.9%) was slightly lower than that in WJH (14.6%). The composition of C, H and N in *Jatropha* hulls changed little (C: 39.2-40.0%; H: 5.5-5.9%; N: 1.1-1.3%) before and after IL-pretreatment.

### Structural characteristics of the recovered biomass

**Figure 1:**
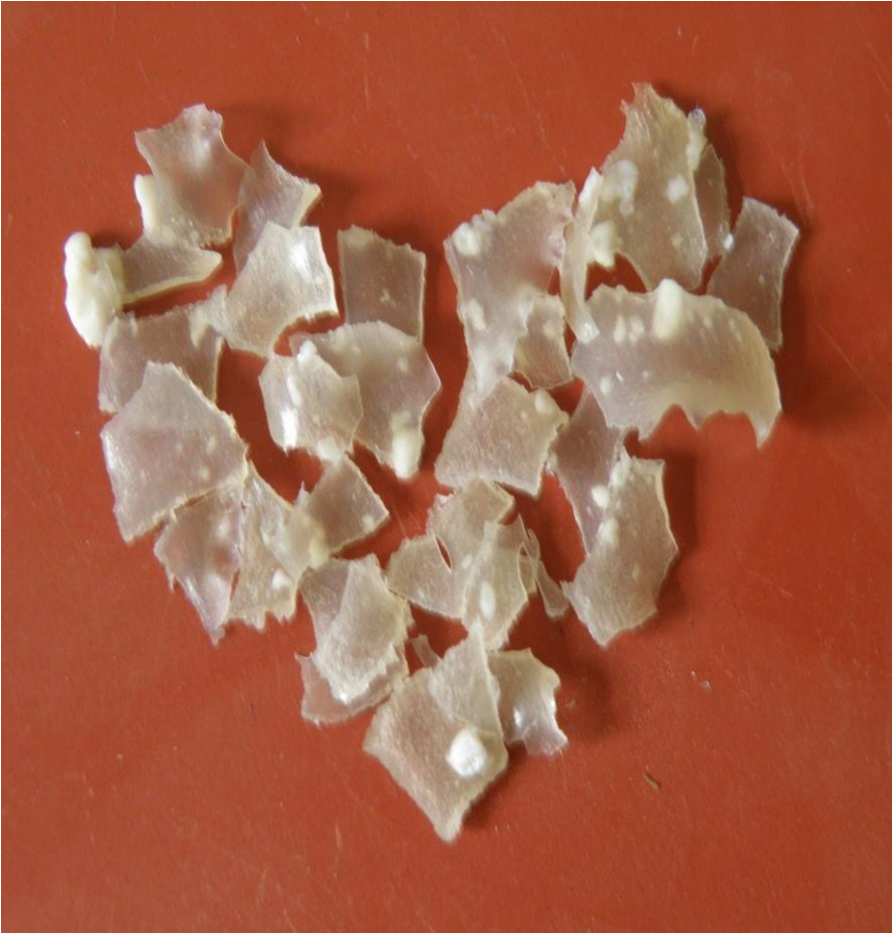
Image of oven dried IL-pretreated cellulose.

**Figure 2:**
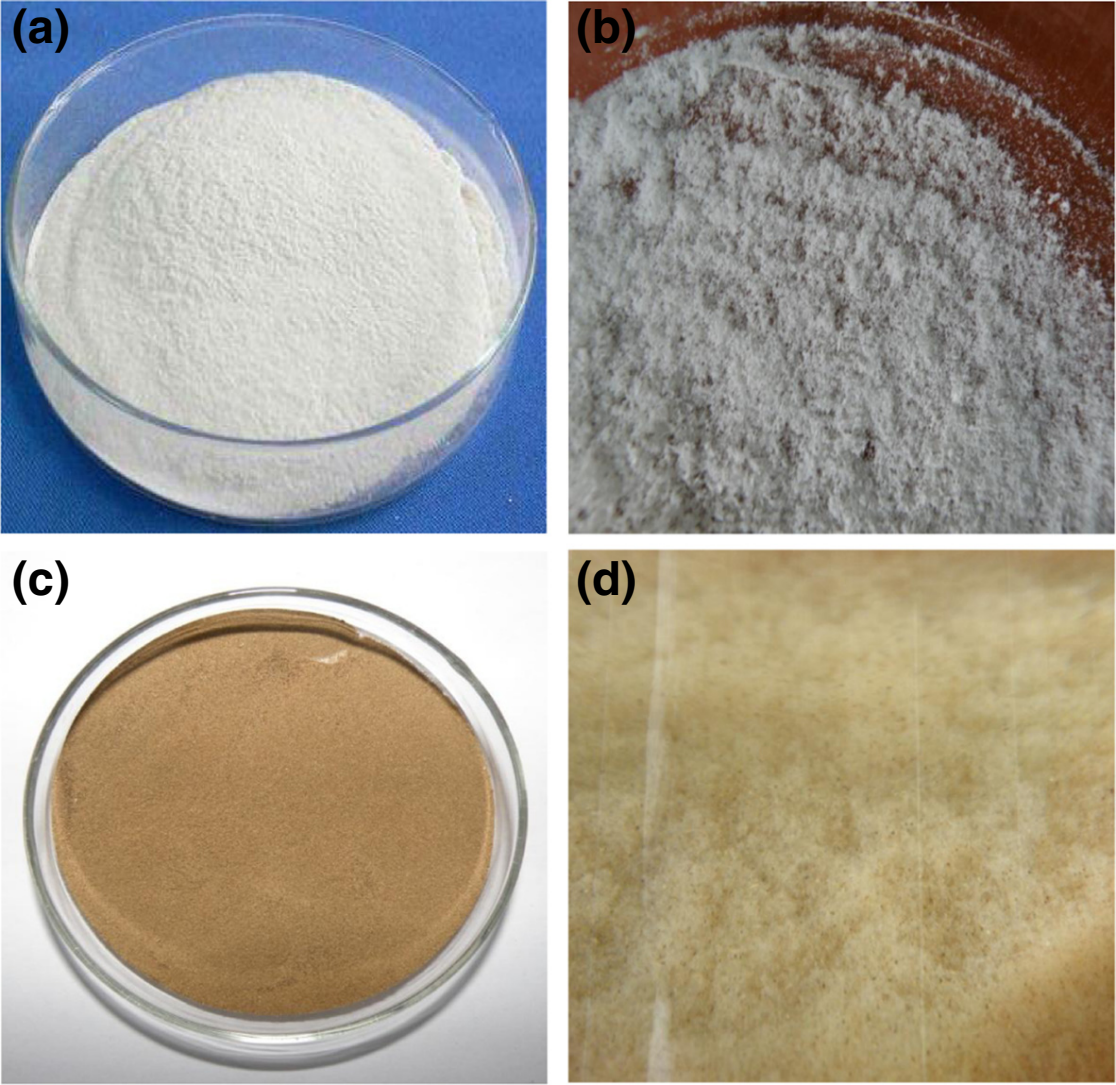
**Images of original and freeze-dried pretreated biomass samples. (a)** Untreated cellulose; **(b)** IL-pretreated cellulose; **(c)** Orginal *Jatropha* hulls; **(d)** IL-pretreated *Jatropha* hulls.

**Figure 3:**
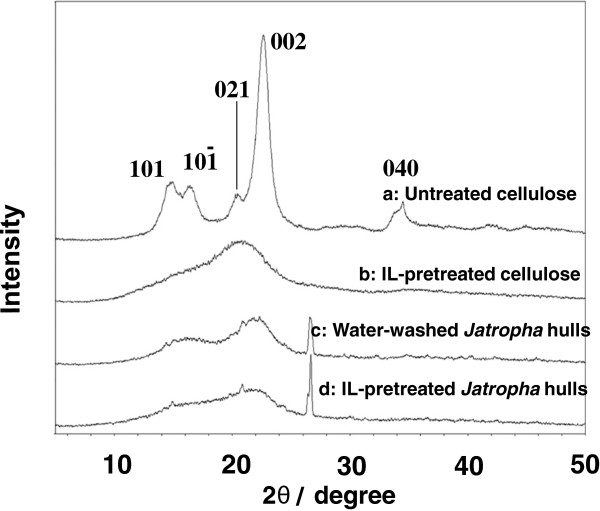
X-ray diffraction (XRD) analysis of biomass samples.

IL-pretreatment could change the crystalline structure and morphology of cellulose by disrupting inter- and intra-chain hydrogen bonding. In this study, after IL-pretreatment and dried in an oven at 70°C, cellulose became hard plastics-like material (Figure 1) that is hard to be hydrolyzed. Therefore, freeze-drying was used and the structure of biomass became fluffy (Figure 2: b *vs.* a; d *vs.* c). The crystallinity and surface area of biomass were evaluated as the most important factors to interpret the structural evolution of biomass after pretreatment. XRD profiles of biomass (Figure 3) showed that untreated cellulose (curve a) has five distinct peaks with their diffraction angles (2θ) at around 14.8°, 16.3°, 20.5°, 22.7° and 34.5° that are regarded as characteristics of plane (101), (10$\overset{-}{1}$), (021), (002) and (040) of cellulose I (Cheng et al. 2011). However, after IL-pretreatment, the intensity of crystalline peaks at 14.8°, 16.3°, 22.7° and 34.5° decreased remarkably or even disappeared with a flat and broad peak remained at around 20.7° (curve b). This was due to the reduction in crystalline degree and the transformation of cellulose I to II (Park et al. 2010). In curve c, the intensity of crystalline peaks of cellulose I in WJH decreased. The reason was that amorphous cellulose, and cellulose connected with hemicellulose and lignin in WJH decreased the crystallinity. After IL-pretreatment, the intensity of peaks (e.g., 002) dropped further (curve d).

CrI of biomass samples was calculated based on XRD data for quantitative comparison (Table 2). The CrI values of untreated cellulose and WHJ were 95% and 56%, respectively. After IL-pretreatment, the CrI value decreased to 18% for ILC and to 19% for ILJH, respectively. BET surface area of recovered biomass samples was analyzed and given in Table 2. The surface area of ILC was 3.7 m^2^/g, 1.54 times that of untreated cellulose (2.4 m^2^/g). Similarly, the surface area of ILJH was 4.4 m^2^/g, 1.52 times that of WJH (2.9 m^2^/g). The significant decrease in CrI and increase in BET surface area would increase cellulose surface accessibility and theoretically enable more efficient hydrolysis.

**Table 2:** Crystallinity index and BET surface area of biomass

**Biomass**	**Crystallinity index (%)**	**BET surface area ( m**^**2**^**/g )**
Untreated cellulose	95	2.4
ILC	18	3.7
WJH	56	2.9
ILJH	19	4.4

### Dilute sulfuric acid-hydrolysis

Based on the previous two-step hydrolysis work on *Jatropha* hulls (Jiang et al. 2012), the mild first-step optimal conditions (150°C, 1.5 wt% acid concentration, 0.5 h) were selected in this work for the hydrolysis of *Jatropha* hulls (WJH and ILJH) to avoid secondary decomposition of sugars from hemicellulose. The severe second-step optimal conditions (150°C, 4 wt% acid concentration, 1 h) were used for the hydrolysis of cellulose (untreated cellulose and ILC). The yields of water-soluble products, reducing-sugars and by-products (5-HMF and furfural) were listed in Table 3. The hydrolysis efficiency of cellulose for sugars production was greatly enhanced after IL-pretreatment. Compared with original cellulose, after IL-pretreatment of cellulose, the yield and concentration of reducing-sugars increased by 139% (from 33.6 to 80.2%) and 139% (from 15.1 to 36.1 g/L), respectively, and the yield of water-soluble products increased by 128% (from 39.5 to 90.0%) after hydrolysis at 150°C with 4 wt% H_2_SO_4_ for 1 h. More cellulose (15 g) was used to obtain high-concentrated sugars, both yield (from 28.7 to 71.2%) and concentration of reducing-sugars (from 21.5 to 53.4 g/L) increased by 148%, and the yield of water-soluble products increased by 128% (from 34.8 to 79.3%). As ILC increased from 9 to 15 g, both the yield of reducing-sugars and yield of water-soluble products decreased to 71.2% and 79.3% from 80.2% and 90.0%, respectively, while the concentration of reducing-sugars increased remarkably (from 36.1 to 53.4 g/L). In the hydrolysis of *Jatropha* hulls, 15-g samples were used in order to obtain high yield of reducing-sugars. Compared with WJH, for ILJH, the yield and concentration of reducing-sugars increased by 80% (from 47.6 to 85.7%) and 76% (from 22.6 to 39.7 g/L), respectively, and the yield of water-soluble products increased by 70% (from 35.5 to 60.2%) after hydrolysis at 150°C with 1.5 wt% H_2_SO_4_ for 0.5 h. The yield of water-soluble products for *Jatropha* hulls was lower than that for cellulose due to its content of lignin (Eq. 4).

**Table 3:** The yields of water-soluble products, reducing-sugars, 5-HMF and furfural as well as concentration of reducing-sugars

Biomass*	Yield of reducing-sugars (%)**	**Concentration of reducing-sugars (g/L)**	**Yield of water-soluble products (%)**	Yield of 5-HMF (%)**	Yield of furfural (%)**
C-9	33.6	15.1	39.5	0.4	0.1
C-15	28.7	21.5	34.8	0.4	0.1
ILC-9	80.2	36.1	90.0	1.3	1.5
ILC-15	71.2	53.4	79.3	0.7	0.7
WJH-15	47.6	22.6	35.5	0.4	1.7
ILJH-15	85.7	39.7	60.2	0.6	2.4

Hydrolysis conditions: 200 mL 4 wt% H_2_SO_4_, 150°C, 1 h for cellulose; 200 mL 1.5 wt% H_2_SO_4_, 150°C, 0.5 h for *Jatropha* hulls.

*C-9: 9 g untreated cellulose;

C-15: 15 g untreated cellulose;

ILC-9: 9 g ionic liquid pretreated cellulose;

ILC-15: 15 g ionic liquid pretreated cellulose;

WJH-15: 15 g water-washed *Jatropha* hulls;

ILJH-15: 15 g IL-pretreated *Jatropha* hulls.

** Data (%) were based on total mass of cellulose and hemicellulose in substrates.

Furfural and 5-HMF were inhibitors in fermentation, while their formation also meant the loss of fermentable sugars. So, furfural and 5-HMF should be avoided. In this work, the increase of the total yield of furfurals (5-HMF and furfural) after IL-pretreatment meant more secondary degradation (from 2.1% for WJH to 3.0% for ILJH). The by-products can be reduced by using more moderate reaction conditions (such as, lower H_2_SO_4_ concentration, shorter reaction time and lower temperature).

### Fermentation

**Figure 4:**
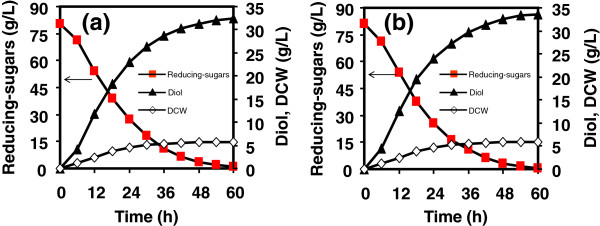
**2,3-Butanediol fermentation by *Klebsiella oxytoca *for 60 h using cellulose hydrolysate as substrate in flasks. (a)** hydrolysate from cellulose, **(b)** hydrolysate from IL-pretreated cellulose.

**Figure 5:**
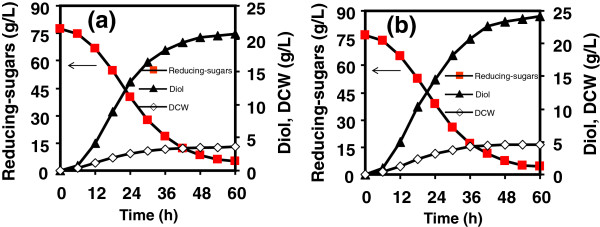
**2,3-Butanediol fermentation by *Klebsiella oxytoca *for 60 h using *Jatropha *hulls hydrolysate as substrate in flasks. (a)** hydrolysate from water-washed *Jatropha* hulls, **(b)** hydrolysate from IL-pretreated *Jatropha* hulls.

After distilled condensation and detoxification by charcoal adsorption, furfural and 5-HMF were removed. The hydrolysates (76.91-81.15 g/L reducing-sugars) were fermented for 2,3-butanediol production, with results given in Figures 4 and 5, and Table 4. In Figure 4a, the fermentation of hydrolysates from untreated cellulose was undertaken with initial reducing-sugars concentration of 80.65 g/L in flasks. After 60 h, 5.76 g/L DCW and 32.41 g/L diol were achieved, giving diol productivity of 0.54 g/(L · h) and yield of 40.61% (equivalent to 81.22% of the theoretical value). In Figure 4b, ILC hydrolysate (81.15 g/L) achieved 5.93 g/L DCW and 33.49 g/L diol, with diol yield of 41.60% (equivalent to 83.20% of the theoretical value) and diol productivity of 0.56 g/(L · h). It was clearly demonstrated that diol yield and productivity from ILC hydrolysate were close to those from untreated cellulose hydrolysate because their hydrolysates had similar components. The hydrolysates from celluloses with and without pretreatment after detoxification were free of the contaminants that inhibited microbial growth and metabolic pathway for 2,3-butanediol production.

**Table 4:** **2,3-Butanediol fermentation by *Klebsiella oxytoca *for 60 h using different substrates in flasks**

**Substrates**	**Diol (2,3-butanediol + acetoin)**			**References**
	**Concentration g/L**	**Productivity g/(L · h)**	Yield* %	
Hydrolysate from cellulose	32.41	0.54	40.61 (81.22)	This study
Hydrolysate from ILC	33.49	0.56	41.60 (83.20)	This study
Hydrolysate from WJH	20.70	0.35	28.60 (57.20)	This study
Hydrolysate from ILJH	24.13	0.40	33.29 (66.58)	This study
Original *Jatropha* hulls hydrolysate from the first-step hydrolysis	4.11	0.069	5.50 (11)	Jiang et al. (2012)
First-step hydrolysate from pretreated *Jatropha* hulls	25.03	0.42	35.6 (71.2)	Jiang et al. (2012)
Second-step hydrolysate from the solid residue of the first step	31.57	0.53	41.4 (82.8)	Jiang et al. (2012)
Glucose	97.4	1.74	49.0 (98.0)	Ji et al. (2009)
Corncob acid hydrolysate	35.7	0.59	50.0 (100.0)	Cheng et al. (2010)
Wood acid hydrolysate	13.3	0.28	29.0 (58.0)	Grover et al. (1990)

*The data in brackets were equivalent of theoretical values.

Figure 5 showed fermentation results of hydrolysates from WJH and ILJH in flasks, with initial reducing-sugars concentrations of 77.41 g/L and 76.91 g/L, respectively. For the fermentation of WJH hydrolysate, 3.58 g/L DCW and 20.70 g/L diol were produced after 60 h, giving diol yield of 28.60% (equivalent to 57.20% of the theoretical value) and diol productivity of 0.35 g/(L · h). For the fermentation of ILJH hydrolysate, products contained 4.61 g/L DCW and 24.13 g/L diol, with diol yield of 33.29% (equivalent to 66.58% of the theoretical value) and diol productivity of 0.40 g/(L · h), which were higher than those of WJH hydrolysate.

## Discussion

In the case of bulk chemicals and biofuels, the cost of the raw material mostly affected the price of the final products. The efficient utilization of biomass was essential for the economical production of 2,3-butanediol. In the previous two-step dilute sulfuric acid hydrolysis of *Jatropha* hulls, total water-soluble products yield was 64%, which was higher than that (37%) from the first-step hydrolysis (Jiang et al. 2012). In this work, the yield of water-soluble products of ILJH reached similar total value (60.2%) by just using the first-step hydrolysis conditions. The yield of water-soluble products for ILJH was greatly enhanced (60.2% *vs.* 37%). In the combination of IL-pretreatment and enzyme hydrolysis of cellulose (Tian et al. 2011), glucose yield was 59% after 72 h hydrolysis time as compared with 80.2% yield of reducing-sugars for ILC in this work. Therefore, this hydrolysis work reached relatively high yield of water-soluble products for IL-pretreated biomass samples under milder conditions.

Separation of 2,3-butanediol from fermentation media is one of economic barriers for the commercial production of microbial 2,3-butanediol (Ji et al. 2011). High concentration of 2,3-butanediol can cut the cost of downstream separation. So, high concentration of initial fermentable sugars is required for practical applications. In the previous study, for example, concentration of glucose (200 g/L) was used and relative high concentration of 2,3-butanediol (95.5 g/L) was achieved (Ji et al. 2009). However, the concentration of total reducing-sugars obtained from lignocellulose hydrolysates was about 20–30 g/L (Guo et al. 2008; Cheng et al. 2010; Jiang et al. 2012). So, increasing the reducing-sugars concentration in lignocellulose hydrolysates is one of the key problems for the high efficient production of biomass-derived 2,3-butanediol. In this work, after IL-pretreatment, the concentration of reducing-sugars increased to 53.4 g/L from 21.5 g/L for cellulose, and the concentration of reducing-sugars increased to 39.7 g/L from 22.6 for lignocellulose.

In the previous work (Table 4), some chemicals after hydrolysis had significantly unfavorable influence on the 2,3-butanediol metabolic pathway and biological activities (Jiang et al. 2012). After fermentation of OJH hydrolysate (obtained from the first-step hydrolysis) for 60 h, only 5.5% diol yield was achieved. However, after washed with neutral detergent to remove extractives (e.g., proteins, lipids, pectins and nonfibrous carbohydrates) and two-step hydrolysis, diol yields reached 35.6% and 41.4% from the hydrolysates of the first- and second-step hydrolysis, respectively. Compared with OJH hydrolysate, the fermentation efficiency of WJH and ILJH hydrolysates were much higher. The reason was that most of fermentation inhibitors produced from the extractives during hydrolysis of OJH were removed by water-washed and IL-pretreatment. IL-pretreatment benefited the fermentation of *Jatropha* hulls hydrolysate more than water-washed pretreatment might due to the more effective removal of extractives. On the other hand, the yields of diol from WJH (28.60%) and ILJH (33.29%) hydrolysates in this work were slightly lower than that from the first-step hydrolysate from neutral detergent pretreated *Jatropha* hulls (35.6%) due to minor extractives still remaining in WJH and ILJH. Glucose had higher efficiency for fermentation than other sugars (Wang et al. 2010). Therefore, the diol yield from the cellulose hydrolysate solution (41.6%) was higher than that from the hydrolysate of ILJH (33.29%), and was close to that of second-step hydrolysate (41.4%) from the solid residue (mainly cellulose) of the first-step hydrolysis of *Jatropha* hulls. Corncob acid hydrolysates were used as feedstocks for fermentation and after 60 h of fed-batch fermentation, a maximal 35.7 g/L 2,3-butanediol was obtained, giving a productivity of 0.59 g/(L · h) and a highest diol yield of 50% reported so far (Cheng et al. 2010). In the work of Grover et al. (1990), wood acid hydrolysate neutralized with Ca(OH)_2_ had been used for 2,3-butanediol production, obtaining 13.3 g/L 2,3-butanediol with a yield of 29% and a productivity of 0.28 g/L h.

In conclusion, in this study, IL-pretreatment and dilute acid-hydrolysis were performed to produce fermentable sugars from cellulose and *Jatropha* hulls. The yield of water-soluble products increased to 90.0% for IL-pretreated cellulose from 39.5% for original cellulose. For *Jatropha* hulls, after IL-pretreatment, the yield of water-soluble products rose to 60.2% from 35.5% for water-washed *Jatropha* hulls. IL-pretreatment also benefited the fermentation of *Jatropha* hulls hydrolysate due to the removal of extractives, with diol productivity increased to 0.40 from 0.35 g/(L · h) for water-washed *Jatropha* hulls. The techniques developed in this paper may be applied to other similar industrial microorganisms for the production of biofuels from biomass wastes.

## Competing interests

The authors declare that they have no competing interests.

## Authors’ contributions

LQJ and ZF (supervisor) conceived the study. LQJ carried out pretreatment, hydrolysis and fermentation experiments, and drafted manuscript. ZF and JL participated in test design and supervision, and revised the manuscript. XKL participated in the preparation of material and hydrolysis experiments and JL participated in the statistics analysis. All authors read and approved the final manuscript.
